# Loss of Prestin Does Not Alter the Development of Auditory Cortical Dendritic Spines

**DOI:** 10.1155/2011/305621

**Published:** 2011-05-15

**Authors:** L. J. Bogart, A. D. Levy, M. Gladstone, P. D. Allen, M. Zettel, J. R. Ison, A. E. Luebke, A. K. Majewska

**Affiliations:** ^1^Program in Neuroscience, Harvard University, Cambridge, MA 02138, USA; ^2^Department of Neurobiology and Anatomy, University of Rochester, Rochester, NY 14627, USA; ^3^Department of Biomedical Engineering, University of Rochester, Rochester, NY 14627, USA; ^4^Department of Brain & Cognitive Sciences, University of Rochester, Rochester, NY 14627, USA; ^5^Center for Visual Science, University of Rochester, Rochester, NY 14627, USA

## Abstract

Disturbance of sensory input during development can have disastrous effects on the development of sensory cortical areas. To examine how moderate perturbations of hearing can impact the development of primary auditory cortex, we examined markers of excitatory synapses in mice who lacked prestin, a protein responsible for somatic electromotility of cochlear outer hair cells. While auditory brain stem responses of these mice show an approximately 40 dB increase in threshold, we found that loss of prestin produced no changes in spine density or morphological characteristics on apical dendrites of cortical layer 5 pyramidal neurons. PSD-95 immunostaining also showed no changes in overall excitatory synapse density. Surprisingly, behavioral assessments of auditory function using the acoustic startle response showed only modest changes in prestin KO animals. These results suggest that moderate developmental hearing deficits produce minor changes in the excitatory connectivity of layer 5 neurons of primary auditory cortex and surprisingly mild auditory behavioral deficits in the startle response.

## 1. Introduction

Early loss of sensory input can have profound effects on the development of sensory cortical areas. Early loss of vision has been shown to affect the development of both inhibitory and excitatory neurons in the visual cortex [1], and trimming of whiskers has similar effects on neurons in somatosensory barrel cortex [2]. While less extensively studied, developmental hearing loss has been shown to induce numerous changes in the response properties of auditory cortical neurons [3]. Sensorineural hearing loss in early postnatal life results in enhanced excitability and weakened inhibition in auditory cortex [4, 5]. Interestingly, even conductive hearing loss, which is a relatively mild deprivation of auditory experience, has similar effects on cortical auditory neurons [6]. 

In visual and somatosensory cortex, excitatory synapses have been shown to be sensitive to sensory manipulation. Manipulations of activity result in changes in the structure and dynamics of dendritic spines [7–11]. These structures are the postsynaptic sites of excitatory connections in the nervous system [12], making them likely substrates for structural plasticity. The shape of dendritic spines has long been thought to have important functional implications [13], and recent experiments have shown that the unique morphology of spines may allow them to compartmentalize calcium and implement synapse-specific plasticity. Thus the detailed morphology of dendritic spines is likely to be crucial for their function. For example, AMPA currents have been found to scale linearly with the size of the spine head [14], while the diameter of the spine neck influences the decay kinetics of intracellular calcium signals [15]. Similarly, spine length has been shown to indicate both the maturity level of a synapse and its potential for plasticity [16–18], while local spine density reveals the relative number of excitatory synaptic inputs onto a section of dendrite [12]. Interestingly, manipulations of the sensory environment have been reported to affect spine morphology, density and dynamics in somatosensory and visual cortices [19–25].

In this study, we asked whether moderate developmental hearing loss affects dendritic spine density and morphology in mouse primary auditory cortex (A1). We used a transgenic mouse model in which knock-out of the *Prestin* gene abolishes somatic electromotility of cochlear outer hair cells [26], increasing auditory-evoked thresholds in numerous subcortical structures by ~40 dB [27, 28]. Despite the moderate loss of subcortically-driven sensory activity, we found no change in the structure and density of dendritic spines along the apical dendrites of layer 5 pyramidal neurons in prestin-null mice. Additionally, the density of puncta of the excitatory marker PSD-95 was unchanged. To test whether behavioral auditory function was altered by prestin loss we carried out behavioral acoustic startle response assays. Interestingly, we found paradoxical increases in acoustic startle responses to moderate, but not high level sounds, suggesting that compensation for sensory loss produces mild hyperexcitability in other auditory centers. This compensation may support the normal development of excitatory synapse structure in primary auditory cortex. Overall, these results suggest that mild developmental hearing deficits do not produce profound changes in excitatory signaling in auditory cortex.

## 2. Materials and Methods

### 2.1. Animals

Prestin wildtype (WT) and prestin knockout (KO) mice [26] were used for assessment of acoustic startle response. For assessment of synaptic characteristics and auditory brain stem responses, prestin KO mice were crossed with Thy-1 YFP-H mice [29] to produce WT:YFP-H mice and prestin KO:YFP-H mice, which express yellow fluorescent protein (YFP) in a subset of cortical layer 5 pyramidal neurons. Genotyping was performed as previously described [26, 29]. All animal work was carried out according to protocols approved by the University of Rochester UCAR committee and the National Institutes of Health.

### 2.2. Auditory Brainstem Responses (ABRs)

Three WT and three prestin KO mice (P30–P35) were anesthetized with a ketamine/xylazine mixture (100 mg/kg/10 mg/kg i.p.). ABR measurements were conducted in a temperature-controlled soundproof chamber maintained at ~32°C. Acoustic stimuli were delivered using a custom assembly consisting of an electrostatic earphone (EC-1, Tucker Davis Technologies) to generate ABR clicks and tone pips. Stimuli were generated digitally (Intelligent Hearing Systems, Smart EP). Needle electrodes were inserted at vertex and pinna and over the bulla, with a ground electrode near the tail. Stimuli were 5-ms tone pips (0.5-ms rise-fall with a cos2 onset, delivered at 30/s) or 100 *μ*s duration click. The response was amplified (10,000x), filtered (100 Hz–3 kHz), digitized, and averaged using Intelligent Hearing Systems SmartEP system. Sound level was raised in 5 dB steps from 10 dB below threshold up to 75 dB SPL. At each sound level, 512 responses were averaged (with stimulus polarity alternated), using an “artifact reject” whereby response waveforms were discarded when peak-to-peak amplitude exceeded 15 V. Upon visual inspection of stacked waveforms, “threshold” was defined as the lowest SPL level at which any wave could be detected, usually corresponding to the level step just below that at which the peak-to-peak response amplitude rose significantly above the noise floor (~0.25 *μ*V). Sound levels were calibrated with a 0.25′′ microphone (Bruel & Kjær model 4136) connected to a measuring amplifier (Bruel & Kjær model 2610).

### 2.3. Tissue Preparation for Histological Analyses

Five wild-type and five prestin KO mice were used, with all animals expressing YFP. Mice were raised until P40 in a normal auditory environment before sacrifice. P40 mice were sacrificed and perfused transcardially with phosphate buffered saline (PBS), and fixed with 4% paraformaldehyde. Brains were extracted, cryoprotected using a 30% sucrose solution, frozen in dry ice, and 50 micron thick sections were collected, mounted, and coverslipped using Prolong Gold antifade reagent.

### 2.4. Fluorescence Confocal Microscopy of Layer 5 Pyramidal Cells

With the aid of a brain atlas [30], areas within the whole extent of primary auditory cortex were identified for imaging on a Zeiss LSM 510 confocal microscope (Carl Zeiss, Thornwood, NY). The distributions of imaged areas within A1 were similar between genotypes. YFP-labeled brain sections were excited at 488 nm and imaged through an HFT 514/633 dichroic and 530 nm–600 nm band pass filter. Excitation power was set to 12% of maximum, and settings for pinhole and detector gain were optimized to minimize photobleaching and utilize the full dynamic range of fluorophore emission intensity. High resolution (512 × 512 pixels) confocal image stacks of layer 2/3 dendritic branches and layer 4 apical trunks were collected using a 100x oil-immersion lens (NA 1.46), at zoom factor 2 (pixel size 0.082 *μ*m), and with a z-step of 0.5 *μ*m. All data were collected with 2x linear averaging and a slow, high quality scan time of 7.86 s per frame. Several additional z-stacks were collected using lower power objectives to document the position of acquired images within the dendritic arbor stacks. Dendritic segments in layer 2/3 were located between 70 and 150 *μ*m from the pial surface and were selected based on the quality of YFP expression and resulting signal-to-noise ratio, so that spines could be identified and measured as accurately as possible. Segments of layer 4 apical trunks were chosen based on distance from the cell body (between 75–150 *μ*m) as well as previously noted image quality considerations. Two regions of interest (each containing 1–5 dendritic sections) were imaged in each layer per section, and three sections were imaged per animal. Sections were processed, imaged, and analyzed blind to genotype.

### 2.5. Analysis of Dendritic Spines

Following data acquisition, z-stacks were exported to TIF format using Zeiss's Axiovision (release 4.6) software. Image analysis was then done using ImageJ (freeware: http://rsb.info.nih.gov/ij/). Z-stacks were filtered using ImageJ's “smooth” function and then collapsed into maximum intensity projections to form 2D representations of 3D dendrites. To quantify spine density, spines were identified by manually stepping through the z-stack, and definite spines were marked on the projected image. Specifically, only spines located in plane with their parent dendritic branch were marked and counted. Spines falling out of plane and those projecting from the parent dendritic branch in solely the z-dimension were systematically excluded from our counts even if they were visually identifiable as spines. After all spines on a segment were marked, segment length was measured using the segmented line tool. 3D segment length was accounted for by measuring the absolute difference in depth between the two ends of the segment and using Pythagoras' theorem. Spine density was then computed as the number of spines per micron of dendrite. We also analyzed the spine dimensions of all dendritic segments located in layer 2/3. Because of the very high spine density of layer 4 dendritic segments, accurate measurements of layer 4 spines proved too difficult to obtain due to highly frequent overlap of adjacent spines. Spine length was measured on maximum intensity projections using a segmented line tool to draw a line from the most distal point of the spine head to the base of the spine neck where it connects to the parent dendritic branch. Measurements of spine head and neck width were made based on fluorescence measurements. The fluorescence profile of a line placed along the center of the head or neck was determined and fit to a Gaussian using custom-written algorithms in MATLAB (The MathWorks, Inc., Natick, MA). Background fluorescence was subtracted before fitting on a dendrite-by-dendrite basis. Great care was taken to avoid saturation in images, and saturated points were removed from the fluorescent profiles. Spines with more than two saturated points were removed from the analysis as it was determined that accurate fits were only obtained if fewer than three points were omitted. This affected a very small proportion of the spines (<3%). The full-width half-max was taken as a measure of spine head width. This method may overestimate the size of small spines that fall under the limit of the resolution of our confocal microscope. The amplitude of the Gaussian fit to the spine neck fluorescence profile was normalized to the amplitude of the fit to the spine head profile as a relative measure of spine neck width.

### 2.6. Immunostaining and Analysis

Fixed, frozen brains (*n* = 3 for each WT:YFP-H and prestin KO:YFP-H) were cut coronally into 50 *μ*m sections, quenched with an H_2_O_2_ solution, blocked in PBS containing 0.3% Triton-X 100 and 5% normal donkey serum (NDS) for 1 hr, and incubated in mouse anti-PSD-95 (1 : 500, Millipore) at 4°C for 48 hrs. Following a 4 hr incubation in Alexa Fluor 594 donkey antimouse IgG (1 : 500, Molecular Probes), the sections were mounted and cover-slipped with Prolong Gold antifade reagent (Molecular Probes). Primary auditory cortex sections were imaged on the Zeiss LSM 510 confocal microscope with a z-step of 0.5 *μ*m, using constant laser intensity and detector gain. For analysis of PSD-95+ puncta density in L2/3 and L4 of A1, a maximum intensity z-projection of 5 slices in the center of the stack was created. After manual thresholding to maximize the projection's signal-to-noise ratio, but retain the lowest intensity puncta, a custom-written ImageJ algorithm was run to count all spots of fluorescence larger than 1 pixel. Because cell bodies tended to partially occlude large areas of our high-resolution images, these areas were measured and subtracted from the overall image area to correct for any puncta that might have been obscured. Density was computed as spot count/corrected area ± SEM. The area of puncta identified in the density analysis was computed to determine the average puncta size. Data were averaged across each animal and condition and computed as average puncta area ± SEM.

### 2.7. Behavioral Assessment of Auditory Function

Prestin KO and WT mice (*n* = 14, 13 resp.) were behaviorally assayed for auditory function via their acoustic startle response (ASR) to brief loud sounds (80–130 dB SPL) [31]. Mice were tested in a wire cage, oval in shape and 5 cm wide, 7 cm long, and 4 cm high. The cage was mounted on a suspended acrylic platform to which an accelerometer was attached directly below the test cage. The cage was placed within a sound attenuating IAC room lined with sound-absorbing foam (inside dimensions ~2 × 2 × 2 m). The force of the startle reflex was transduced by the accelerometer, its signal amplified and routed to an A/D converter, then integrated over a 100 ms period beginning with stimulus onset. ASRs were elicited by 50 kHz broadband noise bursts centered at 25 kHz (25 ms duration including 5 ms rise/fall times) with sound levels 80–130 dB SPL in 10 dB steps. Sound levels were calibrated with a 0.25′′ microphone (Bruel & Kjær model 4135) connected to a measuring amplifier (Bruel & Kjær model 2610). The speaker was mounted directly over the center of the test cage at a distance of 15 cm. The ambient noise level in the chamber was <25 dB SPL for all frequencies above 125 Hz.

### 2.8. Statistical Analyses

Analysis was performed with Prism 5 software (Graph Pad, La Jolla, CA). All values reported in the text are mean ± SEM. For all statistical tests, significance was set to *P* < .05. Mann-Whitney *U*-tests were used to test significance for all morphological measures. Two tailed *t*-tests were used for comparisons of PSD-95 staining. Sample size (*n*) represents individual animals. The behavioral ASR data were analyzed with SPSS 18 (IBM Corporation, Somers, NY), using mixed design repeated-measures ANOVA, with ES Level (80–130 dB SPL) as the within-subjects factor, and genotype (WT or prestin KO) as the between subjects factor.

## 3. Results

**Figure 1:**
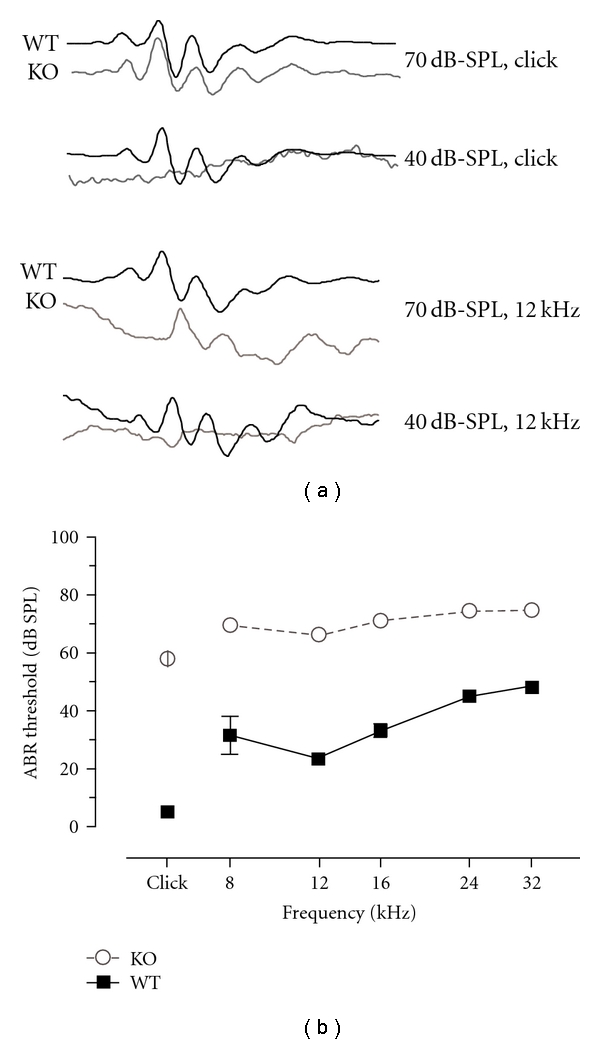
Prestin KO animals have increased ABR thresholds and severely impaired cochlear function. (a) ABR waveforms from WT and prestin KO animals. For 70 dB-SPL stimuli the waveforms are similar in shape, and in both instances (click and 12 kHz) the prestin-null animals have auditory-evoked responses above but not below 40 dB-SPL. (b) Cochlear function is severely impaired in the KO mice with an average threshold shift of ~25–40 dB at all frequencies examined.

**Figure 2:**
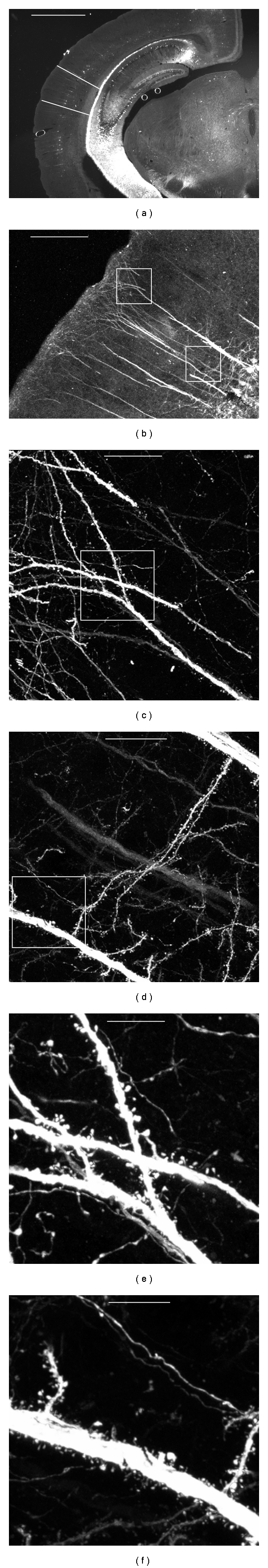
Imaging dendritic structures within primary auditory cortex. (a) Low magnification epifluorescence image of a coronal brain section from a prestin KO mouse. The same section is shown in all panels. A1 was localized at the light microscopic level using a brain atlas. Scale bar: 1 mm. (b) Maximum intensity projection through the thickness of a 10x confocal image stack from A1. Boxes indicate the laminar locations imaged. Scale bar: 200 *μ*m. Maximum intensity projection of a 100x, zoom level 0.7 confocal image stack in layer 2/3 (c) and layer 4 (d). Scale bars: 30 *μ*m. Maximum intensity projection of a 100x, zoom level 2 confocal image stack in layer 2/3 (e) and layer 4 (f). Morphological analysis was carried out on images of this magnification. Scale bars: 10 *μ*m.

**Figure 3:**
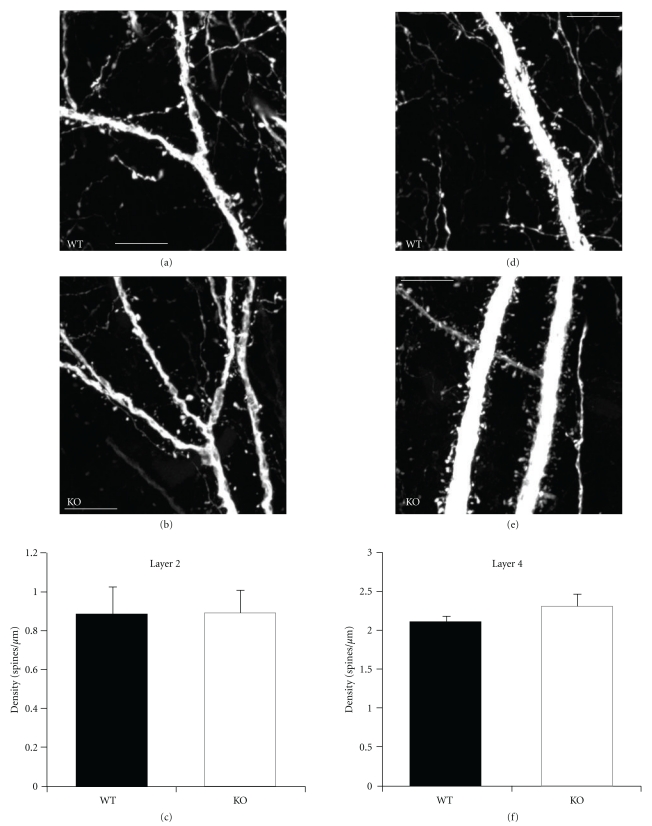
Comparison of spine density in wildtype and prestin knockout animals. Images of dendritic sections in layer 2/3 of A1 of WT (a) and KO (b) mice. (c) Layer 2/3 spine density did not differ significantly between genotypes (*P* > .05). Images of dendritic sections in layer 4 of A1 of WT (d) and KO (e) mice. (f) Layer 4 spine density did not differ significantly between genotypes (*P* > .05). Scale bars: 10 *μ*m.

Developmental sensory loss results in robust changes in excitatory signaling in visual and somatosensory cortices. To determine whether similar changes in excitatory signaling are effected by hearing loss, we examined dendritic spine density and morphology in the primary auditory cortex of prestin KO mice. To examine cortical dendrites we crossed prestin KO mice with thy-1 YFP mice, in which a subset of layer 5 pyramidal neurons are fluorescently labeled. We confirmed that these mice had a deficit in auditory function using auditory brain stem responses (ABRs; Figure 1) at ~P30. Prestin KO:YFP-H mice exhibited ~40 dB increases in auditory thresholds, consistent with previous reports [27, 28]. To assay cortical synaptic changes, we assayed dendritic morphology in young adult mice (P40). We imaged layer 5 pyramidal neurons in primary auditory cortex in fixed brain sections using confocal microscopy (Figure 2). We focused on two dendritic compartments: the superficial apical tuft located in layer 2/3 and the apical trunk in layer 4. While spine density was significantly higher (*P* < .05) in layer 4 apical trunk main branches than in superficial tufts of both genotypes, loss of prestin did not affect the density of dendritic spines in either dendritic compartment (layer 2/3: WT: 0.89 ± 0.14 spines/*μ*m versus prestin KO: 0.89 ± 0.11 spines/*μ*m; *P* > .05; layer 4: WT: 1.88 ± 0.16 spines/*μ*m versus prestin KO: 2.00 ± 0.37 spines/*μ*m; *P* > .05; *n* = 5 animals/genotype) despite the moderate loss of auditory input (Figure 3).

**Figure 4:**
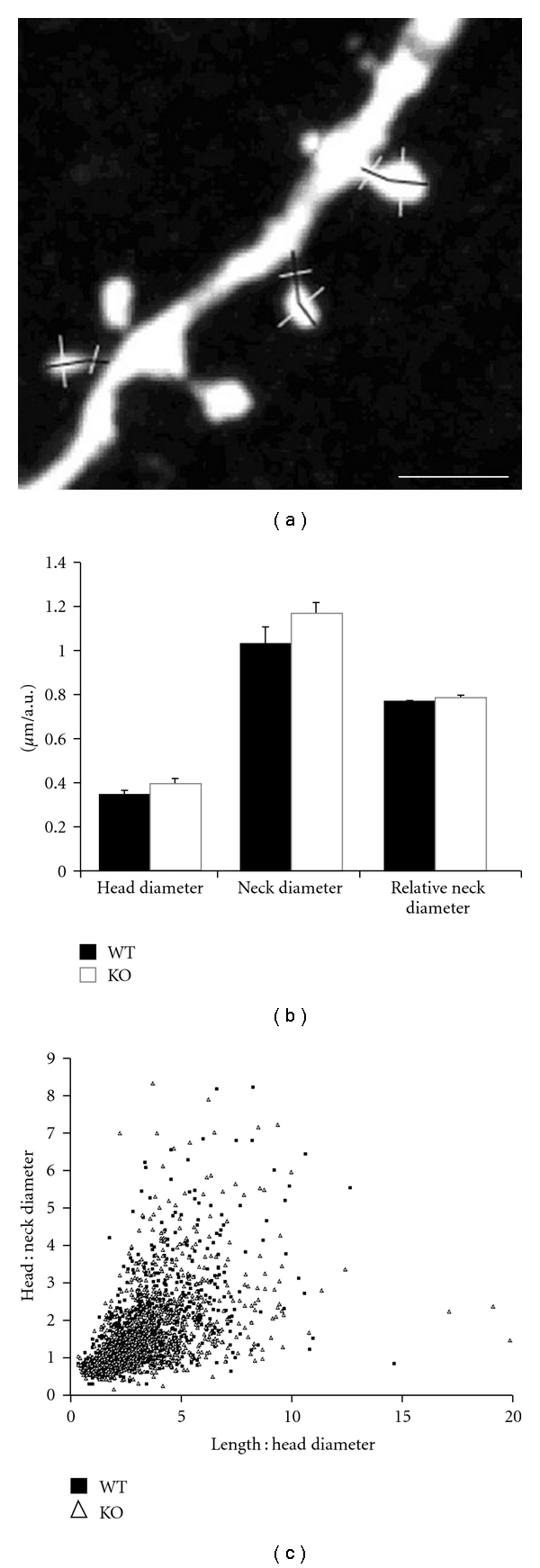
Morphological analysis of dendritic spines. (a) Black lines indicate measurements of spine length. White lines indicate trajectories across which profiles of fluorescence were obtained from the spine head and neck. Scale bar: 2 *μ*m. (b) Measures of spine head diameter, neck length, and relative neck diameter did not differ significantly between WT and prestin KO animals in layer 2/3 (*P* > .05). (c) Spines can be loosely classified into morphological “types” by comparing their relative head, neck, and length dimensions. The complete overlap of WT spines with KO spines indicates that there is no difference in spine type distribution between genotypes.

To determine whether prestin loss resulted in more subtle changes in spine morphology, the dimensions of each spine were measured (Figure 4(a)). We focused on spine length (dendrite to tip of protrusion), spine head diameter (at its widest point), and spine neck width normalized to parent spine (approximate measurement to allow protrusion classification; see Section 2). Only dendrites located in layer 2/3 were considered because the high spine density observed on apical trunks and the brightness of these large dendrites made it difficult to quantitatively assay individual protrusions. In layer 2/3, however, we did not observe any significant differences in dendritic spine morphology in prestin-null animals (Figures 4(b) and 4(c)). Dendritic spine head size was comparable between genotypes (WT: 0.33 ± 0.03 *μ*m, *n* = 1462 spines, 5 animals; prestin KO: 0.39 ± 0.03 *μ*m, *n* = 1387 spines, 5 animals; *P* > .05), as was dendritic spine length (WT: 1.04 ± 0.04 *μ*m; prestin KO: 1.18 ± 0.07 *μ*m; *P* > .05) and relative neck size (WT: 0.76 ± 0.01 *μ*m; prestin KO: 0.79 ± 0.01 *μ*m; *P* > .05). Although dendritic spine morphology exists as a continuum, it has been useful to characterize spines into mature morphologies (e.g., mushroom spines) and immature morphologies (e.g., stubby and thin spines). These criteria are based on the relative proportions of the spine head, spine neck, and spine length [32]. To assay whether changes in the distribution of spines among different morphological types occurred in prestin KO mice, we plotted the ratio of the head to neck diameter against the ratio of the length to head diameter. As expected, spines did not appear to be segregated into distinct categories but rather formed a continuum of different morphologies in both conditions (Figure 4(c)). Additionally, points from WT and prestin KO mice overlapped extensively showing that there was no difference in morphological distribution between the two genotypes. 

**Figure 5:**
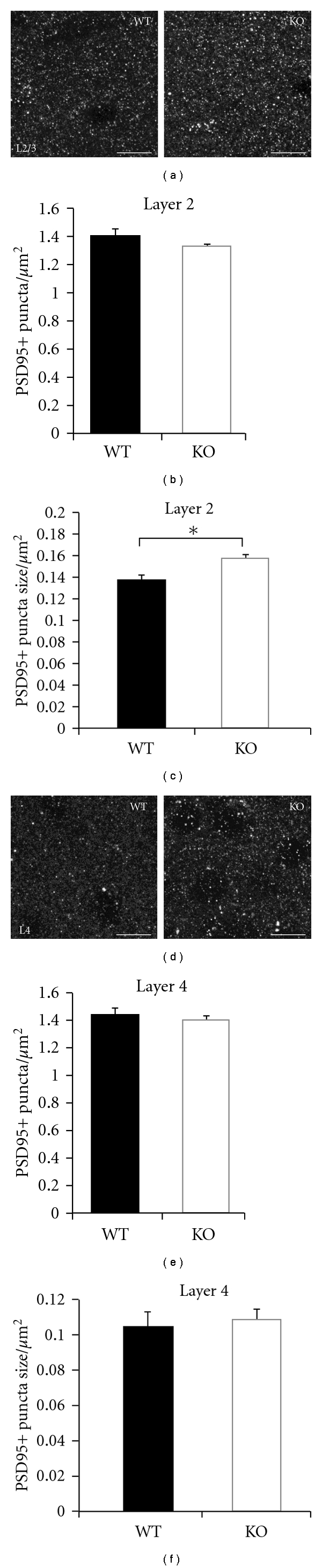
Immunohistochemical analysis of excitatory synapses in wildtype and prestin KO animals. (a) Labeling of puncta positive for the excitatory marker PSD-95 in layer 2/3 of A1. (b) Density of PSD-95+ puncta did not differ significantly between WT (*n* = 3) and KO (*n* = 3) mice in L2/3 of A1 (*P* > .05). (c) The size of PSD-95+ puncta is significantly greater in KO versus WT in L2/3 of A1 (*P* = .015). (d) PSD-95+ puncta in layer 4 of A1. (e) Density of PSD-95+ puncta did not differ significantly between WT (*n* = 3) and KO (*n* = 3) mice in L4 of A1 (*P* > .05). (f) The size of PSD-95+ puncta did not differ significantly between WT and KO mice in L4 of A1 (*P* > .05). Scale bars: 10 *μ*m.

It is possible that changes in cortical excitatory synapse density occur as a result of prestin loss, but that they are not apparent in layer 5 pyramidal cells. To determine whether overall changes in excitatory synapse number occurred in prestin-null mice, we employed a more global method to assay the density of excitatory connections, relying on immunocytochemical staining of PSD-95, a marker of excitatory postsynaptic sites. Fixed brain sections of YFP WT and KO mice were stained for PSD-95, and punctate staining in layer 2/3 and layer 4 was imaged using confocal microscopy. Density of PSD-95 puncta was not significantly different across genotypes in either layer 2/3 (WT: 1.41 ± 0.04 puncta/*μ*m^2^; prestin KO: 1.33 ± 0.01 puncta/*μ*m^2^; *P* > .05; *n* = 3 animals/genotype; Figures 5(a) and 5(b)) or layer 4 (WT: 1.44 ± 0.05 puncta/*μ*m^2^; prestin KO: 1.4 ± 0.03 puncta/*μ*m^2^; *P* > .05; *n* = 3 animals/genotype; Figures 5(d) and 5(e)), further suggesting that mild developmental hearing deficits do not alter excitatory synapse density in auditory cortex. Interestingly, however, the size of PSD-95 puncta was significantly larger in layer 2/3 of KO versus WT mice (WT: 0.138 ± 0.005 *μ*m^2^; prestin KO: 0.16 ± 0.001 *μ*m^2^; *P* = .015; *n* = 3 animals/genotype; Figure 5(c)), while no difference was observed in layer 4 (WT: 0.105 ± 0.009 *μ*m^2^; prestin KO: 0.109 ± 0.006 *μ*m^2^; *P* > .05; *n* = 3 animals/genotype; Figure 5(f)).

**Figure 6:**
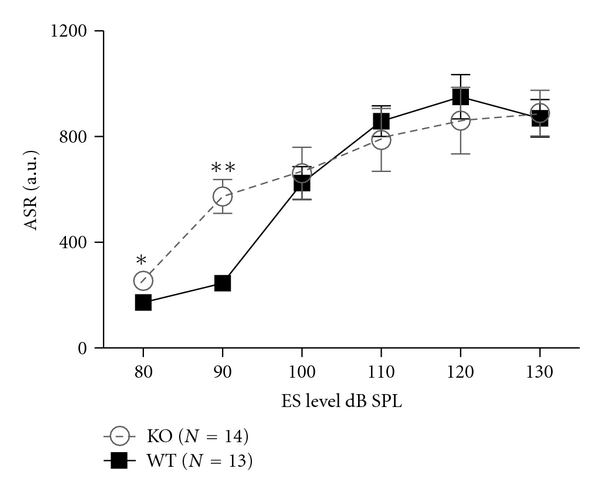
Acoustic startle response in WT and prestin KO animals. The startle response to brief loud sounds increases with sound level for both genotypes, with the prestin KO mice showing significant hyper-excitability at the lower ES levels.

Given the lack of changes in excitatory synapse structure in the primary auditory cortex, we wondered whether compensatory changes occurred upstream of cortical areas facilitating normal cortical development. We reasoned that such changes may also result in less profound deficits in auditory behavior than expected from ABR assays. Therefore, we used a behavioral measure to assess the function of the auditory system after prestin loss. We behaviorally assessed mice via their acoustic startle response (ASR) to brief loud sounds (80–130 dB SPL). Surprisingly, prestin-null mice were hyperreactive to 80 and 90 dB SPL acoustic startle compared with WT littermates, while the two groups responded equally for louder stimuli (Figure 6). Mauchly's test for sphericity was significant (*χ* ^2^(14) = 0.49, *ε* = 0.504), hence the Greenhouse-Geisser correction on the degrees of freedom was used to account for violations of the sphericity assumption. ANOVA indicate a significant main effect of Level (*F*(2.52/125) = 60.3, *P* < .001), and Level x Genotype interaction (*F*(2.52/125) = 4.32, *P* = .011), whereas Genotype was not itself significant (*F*(1/25) < 1). Post hoc ANOVA indicates that the response in prestin KO mice is significantly larger than WT controls at 80 and 90 dB: *F*(1/26) = 7.1, *P* = .014, and *F*(1/26) = 22.6, *P* < .001, respectively; (*P < .05; **P < .001, Figure 1). Paired samples *t*-tests indicate that ASR was significantly above background motor activity for both genotypes at all sound levels: WT *t*(12) > 5.1; KO *t*(13) > 3.4, *P* < .005 showing that both groups of mice have suprathreshold startle responses for 80 dB SPL startle sounds. This result shows that moderate level sounds are coded abnormally in the absence of prestin-mediated cochlear amplification. However the hyperexcitability is particularly mild compared to the more severe effects on auditory-evoked brainstem responses to lower level sounds.

## 4. Discussion

Here we explore the effects of developmental loss of the outer hair cell protein prestin on higher order auditory processing. Our results suggest that moderate developmental hearing loss does not affect the development of dendritic structure of layer 5 neurons in primary auditory cortex. Additionally, density of the excitatory synapse marker PSD-95 was not altered by prestin loss. Surprisingly, despite reduced auditory sensitivity in subcortical auditory centers, prestin null mice show relatively mild hyperactivity in auditory-driven behavior. These data suggest that compensatory changes must exist upstream of cortical auditory centers to ameliorate the auditory deficit in prestin-null mice. These changes may allow normal development of excitatory signaling in auditory cortex and less severe auditory behavioral deficits.

### 4.1. Sensory Deprivation and Dendritic Structure

The development of dendritic structure and excitatory synaptic signaling in sensory cortical areas is highly sensitive to manipulations of the sensory environment, and numerous studies have shown dendritic spine changes in response to sensory deprivation. One of the best described results of sensory deficit is a reduction in the density of dendritic spines in cortical neurons [33] (but see [34]). This has traditionally been thought to be a “trophic” response, whereby a diminished input cannot sustain a large number of target excitatory connections. Alternatively, activity may be needed for synapse maturation. For instance, in the visual cortex, complete removal of visual input by dark rearing results in a delay of development whereby spines adopt an immature phenotype and appear long, thin, and highly dynamic [20, 25, 35]. The appearance of immature and highly dynamic protrusions after deprivation is also a common finding in visual and somatosensory deprivation studies [22, 36–39].

More recently, a different mechanism has been proposed for excitatory changes following sensory deprivation. *In vitro*, reductions in activity result in an upregulation of excitatory signaling through a homeostatic mechanism referred to as synaptic scaling [40]. In synaptic scaling, the neuron senses global levels of activity and scales all synapses multiplicatively to compensate for reduced or enhanced drive, resulting in an optimized firing range and preserved relative synaptic efficacies of all inputs. This effect has been observed in the visual cortex *in vivo* where dark rearing results in augmented excitatory postsynaptic events [41]. Interestingly, monocular deprivation results in similar changes but only during specific critical periods that are lamina-specific [41]. A morphological correlate of this effect has also been described [23]. Dark rearing induces decreases in spine density accompanied by changes in spine shape that include shortening and widening of spines such that the total synaptic area per unit length of dendrite is conserved. Interestingly the changes in spine shape could be reversed by exposure to light while spine density was not, suggesting a dissociation between the mechanisms that govern spine density and spine morphology.

### 4.2. Effects of Sensory Deficits on the Development of the Auditory Cortex

Compared to vision and somatosensation, little is known about the development of auditory cortex, with the majority of studies focusing on the impact of auditory input on the development of auditory brainstem centers [3]. It is clear, however, that auditory input can have a profound effect on the development of auditory cortex as developmental hearing loss induces central deficits that cannot be overcome after restoration of peripheral function [42], and overexposure to a particular tone in young animals results in its enlarged cortical representation [43, 44]. In our study we chose to focus on young adult mice (~P40), as developmental deprivation paradigms have shown changes in synapses at these ages in visual, somatosensory, and auditory cortices [21, 24, 45–48]. At this point, synaptogenesis in auditory cortex is largely complete [49], cortical plasticity subsides [1, 43, 50], and synapse structure and density is likely to reflect the accumulated circuit alterations resulting from altered developmental activity patterns without the interference of adult plastic processes. In fact, in visual cortex, reductions in dendritic spine density during dark rearing are apparent at P25–P48 [19, 48] but normalize thereafter [51] suggesting that early developmental changes may be compensated for in the adult. 

Although inhibitory cortical circuits seem most affected by developmental deafening [52], there are a number of studies that point to altered excitatory signaling in auditory cortex after either deafening or less severe hearing loss. Cortical neurons in auditory deprived animals are more excitable and have altered NMDA receptor signaling [5, 53] similar to those in visual and somatosensory cortices [54–57]. These neurons are also less capable of undergoing long-term potentiation and are more prone to long-term depression [58]. Such altered plasticity could have profound effects on the development of auditory cortex as well as subsequent recovery of function. Additionally, because changes in spine shape and density are associated with long-term plasticity [59, 60], it follows that dendritic spines in auditory cortex are likely sensitive to auditory deprivation. In fact, both unilateral and bilateral neonatal deafening have been previously shown to result in a loss of dendritic spines in auditory cortex [46, 47]. Loss of prestin, however, may result in a different phenotype in auditory cortex than that induced by uni- or bilateral deafening or visual cortical deficits induced by complete loss of vision. Prestin null mice show postnatal deficits (~40 dB threshold shifts; Figure 1) [26], which could be likened to loss of foveal information rather than complete blindness in the visual system. Thus, prestin loss may induce more subtle or specific changes in auditory cortical function than deafening. It is interesting to note that in the visual system, subtle changes in unilateral vision induce more profound remodeling of cortical circuitry than more extreme protocols, suggesting a complex relationship between synaptic alterations and sensory deprivation [61].

### 4.3. Local Changes in Dendritic Spine Function

Given these previous findings, we expected to find changes in layer 5 pyramidal spine density and shape after developmental prestin loss. We assayed two cortical locations on the layer 5 pyramidal neuron dendritic arbor (spines located in layer 2/3 and layer 4) and found no differences between WT and prestin KO mice. One possible explanation for this finding is that the particular spine types we assayed may be part of networks that do not undergo changes in response to sensory experience. However, in visual and somatosensory cortices, layer 5 neurons appear to be particularly plastic, with dendritic spine structure being sensitive to changes in sensory activity [19, 22, 36, 62]. We expected the same to be true in auditory cortex as auditory deprivation leads to decreased excitatory drive to layer 5 cortical neurons [63, 64], suggesting that these neurons may see profound synaptic changes. However, previous studies have described changes in dendritic spine density on layer 2–4 pyramidal cells in auditory cortex [46, 47] making it possible that these are the cell types most affected by auditory deprivation. Such cell and synapse-specific changes have been well documented in the visual and somatosensory cortices, where deprivation causes localized changes within the arbor of specific cell types [36, 62, 65, 66]. To address this issue we assayed the density of excitatory postsynaptic puncta in layers 2/3 and 4. While this approach does not provide synapse identity and morphological information, it does give an overall measure of synapse density that is not limited to synapses on layer 5 cells. As significant differences were not observed in deprived animals, it is likely that synaptic density is not altered by developmental deprivation in these two layers. Analysis of PSD-95 staining in layer 2, however, did reveal changes in the size of PSD-95 puncta possibly suggesting functional changes at other synapses in this layer in the absence of changes in synapse density [67]. Whether such changes are specific to prestin loss or occur with other deprivation paradigms remains to be seen. Our data suggests that excitatory synapses on layer 5 neurons in the auditory cortex may be regulated by sensory activity in a manner distinct from those in other sensory cortices either through effects that do not involve morphological synaptic alterations or that rely on a distinct class of synapses. Because previous studies on excitatory changes in auditory cortex were carried out in gerbils, rats, and rabbits, it is also possible that mouse cortical neurons respond differently to deprivation.

### 4.4. Compensatory Changes in Auditory Function

Prestin KO mice have a large loss of brainstem auditory sensitivity, hence it is surprising that they show rather mild hyperactivity in a behavioral startle assay. This finding, along with a lack of excitatory synaptic remodeling in auditory cortex of these mice, suggests that there may be compensatory mechanisms which counterbalance the loss of sensory drive in the developing auditory system. The locus of these compensatory changes is unclear, but it could occur in lower auditory centers, explaining the lack of effect on auditory cortex. While auditory-driven changes in responses of the inferior colliculus are well documented [3], the acoustic startle reflex is thought to be mediated by the cochlear root nucleus [68], suggesting that compensation may occur upstream of the colliculus, possibly in the form of reduced inhibitory control. This compensation may not necessarily be fully adaptive, as hyperexcitability of acoustic startle previously reported in a mouse model following age-related hearing loss has features akin to tinnitus and hyperacusis [69]. This compensatory remodeling may be specific to milder forms of sensory deprivation, partially explaining the discrepancies between our results and previous studies that have shown dendritic spine remodeling in auditory cortex following developmental deafening [46, 47].
